# 

**Published:** 1920-06

**Authors:** 


					CONTENTS. Page
Operation for the Cure of Prolapse and Cystocele.
By Walter
V-'. Swayne, M.D , B.S.
8i
?n the causes of Death in Lobar Pneumonia.
By F. H. Edgeworth,
M A., m.D., D.Sc.
88
Case of Erythremia or Splenic Polycythemia.
By G. Parker,
M.A., M.D. Cantab.
9i
Th
e Effects of the War on the Mental Condition of the Citizens
Of Bristol.
By R. H. Norgate, M.K.C.S.., L.K.C.F.
95
Pr'table Heart.
A Discussion by Dr. C. F.Coombs, Dr. C. E. K. Herapath,
and Dr. A. G. Morris
io8
r?gress of the Medical Sciences :
Medi
J. M. Fortescue Brickdale, M.A., M.D.
124
Reviews of Books
I27
Editorial Notes
I3?
Library of. the Bristol Medico-Chirurgical Society   133
Meetings of Societies
T35
^ocal Medical Notes
136

				

